# The Association between Emergency Department Overcrowding and Delay in Treatment: A Systematic Review

**DOI:** 10.3390/healthcare11030385

**Published:** 2023-01-29

**Authors:** Adel Darraj, Ali Hudays, Ahmed Hazazi, Amal Hobani, Alya Alghamdi

**Affiliations:** 1 Nursing Department, King Fahad Central Hospital, Health Affairs of Jazan, Ministry of Health, Jazan 82611, Saudi Arabia; 2 Community, Psychiatric, and Mental Health Nursing Department, College of Nursing, King Saud University, Riyadh 11495, Saudi Arabia; 3 Department of Public Health, College of Health Sciences, Saudi Electronic University, Riyadh 13316, Saudi Arabia

**Keywords:** emergency department, time-to-treatment, delay in treatment, accidents and emergency, crowding, overcrowding

## Abstract

Emergency department (ED) overcrowding is a global health issue that is associated with poor quality of care and affects the timeliness of treatment initiation. The purpose of this systematic review is to assess the association between overcrowding and delay in treatment. A systematic review was conducted using four databases (CINAHL, PubMed, Scopus, Cochrane Library), following the preferred reporting items for systematic reviews and meta-analysis (PRISMA). A structured search was conducted to identify peer-reviewed articles aimed at assessing the relationship between overcrowding and delay in treatment, published between January 2000 and January 2021. Only studies that were conducted in the ED settings were included, and that includes both triage and observation rooms. The studies were appraised using two quality appraisal tools including the critical appraisal skills programme (CASP) for cohort studies and the Joanna Briggs Institute (JBI) checklist tool for cross-sectional studies. A total of 567 studies screened, and 10 met the inclusion criteria. Of these studies, 8 were cohorts and 2 were cross-sectionals. The majority reported that overcrowding is associated with a delay in the initiation of antibiotics for patients with sepsis and pneumonia. The review identified that overcrowding might impact time-to-treatment and, thus, the quality of care delivered to the patient. However, further research aimed at finding feasible solutions to overcrowding is encouraged.

## 1. Introduction

Emergency departments (EDs) are one of the most essential departments in the health care system. They provide patients with the required evaluation, stabilization and treatment if needed. General practitioners often refer their patients to the ED for further assessment and investigation when it is impossible to conduct them in primary health care. Therefore, EDs are always used to carry out the initial assessment and admission process, accounting for nearly half of hospital admissions [1]. In 2018, the estimated ED presentations in some of the developed countries, such as the USA and Australia, were 130 million and 8 million patients, respectively [2,3]. The ED unit determines the triage and admission rates, which are some of the valuable measures of the quality of care offered by the hospital [4]. However, due to factors such as a surge in patients, an insufficient number of ED healthcare workers (within 60 min) and lack of resources, the ability to provide timely care is sometimes impeded due to overcrowding in the ED.

ED overcrowding is a recurrent public health concern worldwide [5,6]. Although there is no gold standard definition, overcrowding can be defined as when ED function is impeded because the number of patients waiting to be seen and treated exceeds the physical/staffing capacity of ED [7]. Moreover, when the number of ED staffing is short compared to the high number of patients waiting to be seen, overcrowding occurs. To measure how crowded an ED is, several scoring systems, such as the National ED Overcrowding Scale (NEDOCS) and the National Emergency Access Target (NEAT) were developed to provide a perspective on the problem [8,9]. Variables such as occupancy rate, length of stay (LOS) and boarding time are used to measure how crowded an ED is. Most EDs worldwide are hampered by overcrowding and the number of visits to the emergency services is on the rise. In Australia, for example, in 2018, the Australian Institute of Health and Welfare [2] (AIHW) reported a 3.4% increase in ED visits compared to previous years. Similarly, in the USA, there was a 51% increase in ED visits and a 12% decrease in the number of EDs from 1995 to 2016 [10]. This indicates that more critically ill patients require care in EDs. Therefore, hospitals lack the sustainable capacity to respond to high demand for emergency services besides struggling to equip the ED with the required resources. In addition, overcrowding translates into the physicians’ and nurses’ malpractice of the standard norms, as well as service standards for patient care [11]. This global patient care challenge would consequently hinder quality of care and, therefore, lead to undesirable consequences such as an increased mortality rate, leaving the unit without treatment, waiting for longer hours, and delay in treatment.

Delay in treatment can be described as the period of time from the occurrence of symptoms to the initiation of treatment [12]. Such delay is likely to result in negative consequences and poor outcomes. For example, timely-delivered antibiotics within 3 h or administration of thrombolysis within 60 min are considered standard measures of care quality, thus, a breach in timeliness of delivery could worsen the patient’s progression [13,14]. Moreover, treatment delay is reported to impact ED patients and lead to poor prognosis [15,16]. In its recent published report, AIHW [3] showed a decline in the overall proportion seen on time, with a few patients completing their ED visits within 4 h. The compelling predictors of delays in the treatment process include increased waiting patients due to the high patient volumes, ambulatory diversion, and potential increase in the LOS. Chang et al. [17] established that adverse clinical outcomes are inevitable when physicians and frontline nurses face delays in treatment. The high patient volumes in the ED occasions non-compliance with the appropriate treatment guidelines. Some patients require return visits and readmissions, which increases the period for receiving treatment as well as potential susceptibility to morbidity and death [4]. However, there has been a lack of proper reviews on the effect of ED overcrowding on treatment delays.

Current and recent reviews have focused more on the causes and solutions than emphasizing treatment delays. Carter, Pouch and Larson [18] undertook a systematic review to establish the link between ED crowding and patient outcomes. The analysis of the 11 articles that met the eligibility criteria found that ED crowding caused mortalities during the admission or the discharge phases. The review further associated the ED crowding with the escapes of patients, and increased safety concerns. Morley et al. [19] relied on a larger sample of 102 studies than Carter et al. [18] did to assess the causes and consequences besides the solutions to ED crowding. The analysis found delayed assessment and treatment and high patient volumes as the primary causes of ED crowding. The problem then leads to increased LOS, mortalities, staff malpractice and stress, as well as non-adherence to the treatment guidelines. Reviews of original research have not explored or confirmed the link between ED overcrowding and delay in treatment. For instance, Rasouli et al. [20] relied on 58 articles to examine the multiple outcomes of patients or healthcare-related challenges. The assessment of the healthcare outcomes of the cohort studies found the general effect of ED crowding on the patients, communities, and healthcare systems to be the prevention of efficient delivery of care. ED crowding emerged as a cause for the poor quality of care, inefficiency, and increased operational costs due to excessive patient LOS. Overall, the recent reviews lack proper emphasis on treatment delay, which compels the need for a further systematic review of empirical evidence to determine the link between ED overcrowding and delays in patient treatment.

The findings of the systematic review will be valuable for ED health care workers. They will use the findings to create pathways for the timely assessment of patients needing emergency care. Nurses, for instance, could use the outcomes of this systematic review to adopt early drug administration to enhance patient outcomes. Hospitals might consider proper measures for enhancing prompt treatment to improve the quality of care and reduce the cost related to increase LOS, morbidities and mortalities following ED overcrowding. The review will propose a more effective measure than the existing guidelines to promote the effectiveness of the emergency service care and the subsequent healthcare service delivery affected by the ED efficiency levels. The healthcare system delivery processes should readjust the organization of the EDs to respond to the issues of delaying the delivery of appropriate and satisfactory treatment to different patients.

### 1.1. Aim

The systematic review aims to establish the association between overcrowding and delays in treatment in the emergency departments. The findings are supposed to help decision makers to better understand this problem and, thus, promote prompt solutions.

### 1.2. Search Question

The PEO (population, exposure and outcome) mnemonic was used to frame the search question for the systematic review. The framework assisted in structuring a well-built and focused clinical question to initiate literature research as follows:

P—Emergency department patients
E—Overcrowding
O—Delay in treatment

## 2. Materials and Methods

### 2.1. Search Strategy

A search strategy was conducted on four electronic databases. The databases included PubMed, CINAHL, Scopus and Cochrane Library. The search required the application of keywords derived from the PEO framework question. The keywords included “emergency department”, “emergency service”, “accident and emergency”, “ED,” “overcrowding”, “crowding”, “ED volume”, “delays in treatment”, “casualty”, “accident & emergency department”, and “time to treatment.” The search terms or keywords were combined using the Boolean Operators, “AND” and “OR”. Using different search terms and incorporating the two operators led to a focused search, which then generated the relevant journals in line with the research questions. Furthermore, filters were applied as search limits in the search process to generate the appropriate studies. The filters include studies published between January 2000 and January 2021 as a mean to find relevant and updated studies, scholarly and peer-review studies. Eligibility criteria were established to facilitate the selection of the journal articles. The language was limited to English for practical reasons. The search strategies were designed to be broad in order to find relevant material with high sensitivity.

### 2.2. Inclusion and Exclusion Criteria

The inclusion criteria for selection in this systematic review consisted of studies that are of a quantitative type. This included prospective/retrospective cohorts, cross-sectional studies, and published, peer-reviewed full text articles. Journal articles whose primary outcome is delay in treatment was the main outcome of interest. Participants who are adults (>18 years) were included. Only studies that were conducted in the ED settings were included and that includes both triage and observation rooms. Although PRISMA guidelines [21] indicated that there are no limits to either date or language when searching, language was limited to English for practical reasons. Published studies were limited to between January 2000 and January 2021 to identify updated and relevant studies. Studies published in the last 20 years were considered relevant, including recent literature that was most likely to be currently in use and to reflect the most current conditions in light of the current recommendations for emergency department overcrowding and delay in treatment. Studies that did not address the research question were excluded. Systematic reviews and the pediatric population were also excluded.

### 2.3. Data Abstraction

After including the final acceptable studies for review, data extraction was conducted using a structured table that involves detailed descriptions of the finalized studies, such as the author’s name, date, design, setting, sample size, characteristics of participants, mean age, outcome measure, statistical results and comments (Table S1).

### 2.4. Data Synthesis

A narrative synthesis was conducted using a textual and tabular approach in order to summarize and evaluate the body of evidence included in the review. The integration and interpretation of the results then were reported in the discussion, following the guidelines of Popay et al. [22].

## 3. Results

### 3.1. Search Outcomes

Two independent reviewers (A.Ha and A.Ho) identified potentially relevant citations matching the selection criteria based on the review of the title and abstract. Also, two independent reviewers (A.D and A.A) examined the electronic searches and obtained full reports of all citations that were likely to meet the predefined selection criteria. Data abstraction was performed by two reviewers and verified by two reviewers. Any discrepancies in the screening of titles/abstracts and full-text articles were resolved via discussion, with adjudication by a third reviewer if necessary.

PubMed, Medline and CINAHL generated a total number of 621 articles, which underwent a selection process following the preferred reporting items for systematic reviews and meta-analyses (PRISMA) flow diagram suggested by Liberati et al. [23], as outlined in Figure S1, to describe the screening procedures [21]. Hand searching and snowballing of the identified articles were conducted in order to find any missing papers. The selection process then began with the elimination of the duplicates from the records gathered from PubMed, CINAHL, Scopus and Cochrane Library via EndNote X7 software. Then, after the initial review of titles and abstracts, non-relevant articles were removed. 145 full articles were left, however, 135 were excluded as they did not meet the inclusion criteria. Finally, a total of 10 articles were included in the review, as shown in Figure S1 and Table S1 (Supplementary Materials).

### 3.2. Quality Appraisal

For the selected studies, two quality appraisal tools were used: the CASP checklist tool for cohort studies and the Joanna Briggs Institute (JBI) checklist tool for cross-sectional studies. The quality assessments of all papers were independently assessed by two reviewers (A.D and A.H). Disagreements were resolved by consensus and after discussion with a third reviewer. The critical appraisal skills programme (CASP) tool for the cohort studies was used to assess the identified cohort articles [23] (Table S2), while the JBI checklist tool was used to appraise the quality of the two cross-sectional studies [24,25] (Table S3) (Supplementary Materials) Both the CASP and JBI tools indicated that studies are required to meet all the questions in the checklist. For cohort studies, none of them fulfilled all of CASP checklist criteria. Four studies [25,26,27,28] failed to address the potential confounders whereas the other six studies [24,29,30,31,32,33] could not provide enough detail as to whether possible confounders were controlled or corrected. Similarly, the two cross-sectional studies failed to acknowledge all the confounding factors. Hence, this uncertainty may play a role in the cause-and-effect relationship. The level of statistical analysis was reasonable, with the presence of confidence interval (CI 95%) in the results reported except for the following studies [25,29,30,31], in which CI 95% was absent. In addition, participant follow-up was absent in most studies, which could result in bias.

### 3.3. Study Characteristics

The studies included were published between 2000 and 2021. Nine of them were from the USA and one from the Netherlands. Two studies were cross-sectional [24,25] whereas eight were cohorts; seven retrospectives [26,29,32] and one prospective [31]. Various settings were represented, community (*n* = 3), academic (*n* = 7) and trauma (*n* = 2). The sample size had a total number of 117,996 participants, which ranged from 334 to 39,110 (median 3242). Participants in the included studies were mostly comprised of patients with community-acquired pneumonia (CAP) [24,25,32,33] and sepsis [33,34,35]. Other studies presented patients with abdominal pain [31], back pain [29] and general pain [26,30]. Studies were conducted in various settings with academic centers being the highest proportion. Effect modifiers that were considered relevant were triage acuity score, race and sex. All studies fully addressed both exposure and outcome measures. For exposure measure (ED overcrowding), the following were used as indicators; occupancy rate (percentage of ED beds filled), total patient-care hours, number of patients in the waiting room and number of admitted patients boarding in the ED. In regard to outcome measure, studies in this review examined outcome associated with delay in antibiotic treatment and delay in analgesia administration.

#### 3.3.1. Time-to-Antibiotics

Six studies evaluated the impact of ED overcrowding on the timeliness of antibiotics. Of those, four studies were measured from triage (ED arrival) and two were measured from ED registration (room placement). Two studies [34,35] examined the exposure measure on patients who presented to the ED with sepsis and reported a significant delay in the administration of antibiotics of more than 3 h, despite the international guidelines that recommends initiation time within 1 h [36]. They recorded that occupancy rate was the primary crowding measure that caused delay in antibiotics administration as for each 10% increase in the number of patients, there was a 5-min delay. Gaieski et al. [28] reported that the effect of crowding on the initiation of antibiotics within 1 h in day shift was more significant than that of night shift [OR 0.73 (95%CI 0.59–0.92); OR 0.46 (95% CI 0.51–0.81), respectively].

Four studies examined treatment delay from room placement for patients presenting with CAP. Of those, two found that high occupancy rate was the primary predictor [24,33] while the other two reported LOS and waiting room to be the primary predictors [25,32]. Similarly, all studies reported delay in antibiotics therapy, despite the recommended guidelines of within 4 h [37]. It was also reported that neither change of seasons nor shift time period played a major role in the impact of overcrowding on treatment delay [24,33]. In addition, some medical conditions, such as CAP, are highly likely to require further investigation in order to confirm the diagnosis, which consequently increased processing time, scarce resources and provided a less efficient diagnosis [25,32].

#### 3.3.2. Time-to-Analgesia

Three studies reported that delay in triage-to-analgesia was likely to be more significant than that of room placement [26,29,31] in which the time median range of administration ranged from 107–130 min, exceeding the 1 h target as shown in Table S4 (Supplementary Materials). One study found that poor assessment and treatment was the result of high patient inflow which led to delay in analgesia administration [26]. All studies confirmed the impact of all ED crowding measures on treatment delay and, moreover, one study indicated that delay may occur even in the presence of a low occupancy rate [30].

#### 3.3.3. Crowding Measures

Total six of ten studies reported that occupancy rate and waiting room were the highest crowding measures responsible for treatment delay [26,29,31,33,34]. They estimated that when ED occupancy rate is at the highest level, the probability of administering medication at the right time is 50% lower than when occupancy rate is adjusted. Several factors can impact such delay when occupancy rate is at its peak. For instance, nursing staff are likely to start the assessment and treatment plan to the existing patients, thus neglecting the ones who need earlier treatment [33]. Similarly, when nurse-to-patient ratio is unmatched, especially in the ED, the likelihood of receiving a timely-treatment is minimal [29]. On the other hand, two studies reported that LOS was the primary predictor of treatment delay [25,30] in which the *p* value was <0.001 and 0.04, respectively.

#### 3.3.4. Patient Care Process

Various studies reported consequences related to transition of care process as a result of ED overcrowding. Two studies [33,35] reported patients presenting to the ED with sepsis encounter major delay in the initiation of protocolized care, primarily due to the fact that a medical condition such as sepsis requires multiple nursing/medical interventions prior to the initiation of antibiotics [36,37]. Similarly, Mills et al. [32] and Pines & Hollander [27] stated that as ED becomes crowded, existing patients with acute pain such as abdominal or back pain experience delay in the progress of their assessment. This is mainly because such cases demand ED physicians to postpone analgesia administration until they exclude secondary causes.

They suggested that tasks such as initial assessment, a physician’s examination, diagnostic procedures (blood test, ECG, imaging) are key factors that are likely to contribute to treatment delay. Interestingly, one study [25] found that boarded patients experienced major delay in their care process, however, those who need ICU bed are less likely to wait longer and the probability of admission is higher than those who need admission to general units.

#### 3.3.5. Staff Adherence to Guidelines

Poor adherence to treatment guidelines was reported to be associated with overcrowding. Delay in initial assessment and prompt administration of analgesia was reported in two studies [27,30]. Similarly, four studies showed that as ED crowding increases, HCWs compliance to the implementation of protocolized care was negatively impacted [25,26,30,35].

## 4. Discussion

This systematic review provides a detailed summary of the impact of ED overcrowding on patients’ outcomes in terms of delay in treatment. The results of this review show that patients of different medical conditions and acuity were affected by the fact that they have to wait for a longer time until medication is administered during high ED inflow. This would consequently impact the process of care being delivered to the patients. To the best of the authors’ knowledge, this is the first systematic review to focus on delay in treatment during ED overcrowding. Previous reviews [16,17,18,19,20] have drawn attention to the possible causes and solutions of ED overcrowding. However, neither one of them have stated explicitly how overcrowding could be associated with delay in treatment, particularly time-to-antibiotics and time- to-analgesia. The fact that these reviews have failed to address issues to do with delayed treatment means there is a need for a systematic review using the empirical evidence available. Determining the reasons behind overcrowding in ED and the delayed treatment that results is a good way to find recommendable solutions.

This review highlights the fact that delayed treatment will potentially lead to the departure of patients from ED without being seen. This walkout often occurs simply because some patients cannot wait because of increased LOS. Not only this, but delay in treatment can also increase morbidity rate and consequently, mortality rate. In addition, failure to comply with prompt administration will significantly impact patient satisfaction, and when the rate of satisfaction is low, stress and violence between patients and HCWs may arise. Moreover, some settings may have a limited number of beds. As a result, ED clinicians are more likely to discharge patients who still require further assessment and diagnostic procedures or even with high-risk medical conditions. Inevitably, morbidity and mortality rate would increase. Several studies [38,39,40,41] reported that for every 1-h delay in the initiation of antibiotics for patients with sepsis, there is a higher chance of hospital mortality, which confirms the findings of this review. In addition, one study [42] identified multiple organ failure in septic patients as for each one-hour delay. In a study that investigated the impact of delayed antibiotic administration in patients with CAP, it found that not only delay can result in treatment failure, but it also can lead to LOS and increased treatment cost [43]. In contrast, prompt administration is highly likely to improve patient outcomes. Studies found that the initiation of antibiotics within the targeted guidelines can significantly improve patient outcome and, thus decrease mortality rate [44,45]. Therefore, delay in treatment as a result of ED overcrowding is a major public health concern that contributes to negative ramifications.

Existing reviews such as Morley et al. [19] and Rasouli et al. [20] only mentioned the studies that proved there is a link between ED overcrowding and poor patient outcome, however, they were unable to discuss the statistical findings and their implications in that regard. This review provides an in-depth thematic analysis and synthesis that can contribute to finding future solutions to the problem. Moreover, this review unraveled the primary crowding measures that affected the timeliness of treatment provided to the patients while previous reviews failed to find a connection between them.

The systematic review confirmed that HCWs such as nurses and physicians are prone to breaching the standard norms when attending to patients, in a move to treat the large number of people who might be requiring services. Overcrowding in ED results in a scenario where quality care is missed in this sensitive hospital department, based on the critical cases handled. It was established that a significant number of sepsis patients have ended up waiting for more than 4 h to receive the initial antibiotic treatment. Such delays have a critical impact on the patients’ outcomes, considering that sepsis is a life-threatening condition. The delayed application of the standard guidelines worsens the condition of an affected patient such that the healing process is delayed, while death can occur in critically ill patients [46]. The poor prognosis associated with overcrowding in ED is a form of medical and nursing malpractice that leads to poor patient outcomes thereafter.

The fact that most hospitals lack the sustainable capacity to respond to the high demand for emergency services is a health issue that requires a great deal of input to solve. Stakeholders in the healthcare sector have the task of liaising with the government to address some of the factors known to cause overcrowding in ED. Staffing issues are factors that the state and the federal government can address by employing more qualified HCWs to reduce the workload in the ED. Shortage of nurses is an issue that has remained a burden, but it is important that the authorities responsible make efforts to increase the number of nurses working in ED and other departments dealing with critically ill patients. It is important that workable solutions to staffing issues, holding capacity of ED, reliability of ambulatory services, and right medical equipment are developed and put into practice [21]. Adherence to the standard operating procedures should act as a guideline when developing and implementing such solutions.

The review indicates the need to reorganize the processes involved in ED to help reduce overcrowding cases that have been reported in various healthcare facilities. In this case, reorganization is a way to make informed changes to the existing processes in the ED to address the most important issues that are worsened by overcrowding. The reorganization should push for the establishment of a special team of HCWs to attend to terminally ill patients who should be managed promptly.

### 4.1. Strengths and Weaknesses

The systematic review identified common issues leading to overcrowding in ED and revealing the experiences of patients. The review is an important one in that it has been carried out at a time when there are limited studies which highlight issues related to delayed treatment. However,, the review fails to account for workable solutions that have currently been used to reduce overcrowding in some facilities. It only gives a generalized overview of solutions that can be used. It is recommendable that patients should be properly screened at the reception to identify the severity of their medical condition to determine whether they should be considered for urgent care or can wait if their condition does not worsen. Improving ambulatory services is a recommendable way through which health facilities can make a referral for terminally ill patients who cannot be kept waiting.

The systematic review confirmed the effects of overcrowding that were contained in the previous studies. The review identified the delayed administration of antibiotics and analgesia, increased length of stay, high occupancy rate, increased time of treatment from triage to room placement as the main effects of overcrowding in the emergency department.

### 4.2. Limitations

The review included studies published in only English, which might lead to the overlooking of non-English studies. As a result, studies published in other languages are likely to provide a broader perspective. In addition, all of the included studies were conducted in high-income countries, and key factors such as cultural, socioeconomic, and available health resources vary between developed, developing, and underdeveloped countries, which can impact the study validity. Furthermore, although observational studies can assist in identifying the probable cause and effect association, the ability to exclude potential confounders is strenuous. Furthermore, the studies included in this systemic review examined delays in antibiotic treatment and analgesia administration only, while there are other delayed treatments, for instance the administration of thrombolysis possibly associated with emergency department overcrowding, and they were not examined. Thus, it is an important topic that needs further research in future.

## 5. Conclusions

This quantitative systematic review analyzed the literature on the association between ED overcrowding and delay in treatment with much emphasis on the timeliness of administration. The review highlighted two outcomes that are impacted by overcrowding: time-to-analgesia and time-to-antibiotics. A number of crucial themes were identified and synthesized in relation to the impact of ED overcrowding. Nevertheless, there were some gaps in the review that could affect its results. Therefore, to fully understand the relationship between ED overcrowding and treatment delay, more comprehensive and rigorous studies are recommended. It is also imperative that health organizations address these issues and endeavor to promote safe practice and optimal quality of care that can be delivered by HCWs to their patients.

The findings of the systematic review will be valuable for ED health care providers. They will use the findings to create pathways for timely assessment of patients needing emergency care. Nurses, for instance, could use the outcomes of this systematic review to adopt early drug administration to enhance patient outcomes. Hospitals might consider proper measures for enhancing prompt treatment to improve the quality of care, reduce the cost related to increase LOS, morbidities and mortalities following ED overcrowding. The review proposes a more effective measure, along with the existing guidelines, to promote the effectiveness of emergency service care and the subsequent healthcare service delivery affected by the ED efficiency levels. The healthcare system delivery processes should readjust the organization of the EDs to respond to the issues, which cause delay in the delivery of appropriate and satisfactory treatment to different patients.

## Data Availability

Data is contained within the article.

## Supplementary Materials

The following supporting information can be downloaded at: https://www.mdpi.com/article/10.3390/healthcare11030385/s1, Figure S1: PRISMA Flow Diagram for Study Selection; Table S1: Characteristics of included studies; Table S2: CASP checklist for cohort studies; Table S3: JBI checklist for cross-sectional studies; Table S4: Delay of analgesia as recorded by median time.
