# Supplementary material for: The Association between Emergency Department Overcrowding and Delay in Treatment: A Systematic Review
Source: Healthcare (Basel). 2023 Jan 29;11(3):385. doi: 10.3390/healthcare11030385 (PMC9914164; doi:10.3390/healthcare11030385)
Supplement: Supplementary file 1 [file healthcare-11-00385-s001.zip › healthcare-2137553-supplementary.pdf]

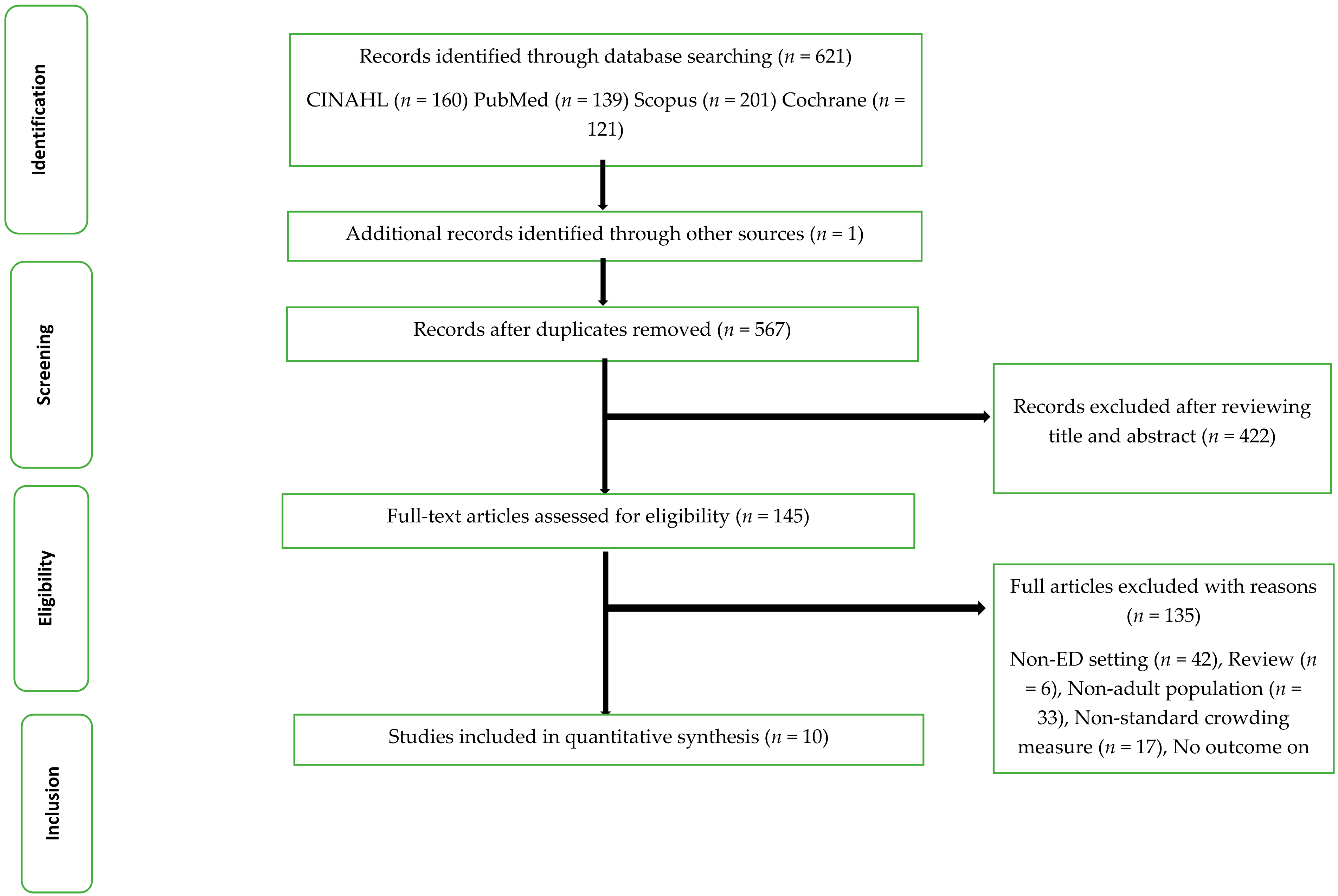

Figure S1. PRISMA Flow Diagram for Study Selection (Moher, Liberati, Tetzalff, Alt-man, 2009).

Table S1. Characteristics of included studies.

Author	Design	Setting	Sample size	Participants	Outcome measure	Statistical results	Comments
**Fee et al (2007)**	Cross- sectional	USA, university hospital Jan 2004-June 2005	486 adults, mean age: 68 yrs, 53% male	patients with CAP	Antibiotic delivery within 4hrs	61% received antibiotics within 4hrs; 92% within 8hrs, OR 0.96 (95% CI 0.93 to 0.99)	for each additional regular patient arrival, CAP patients chance of receiving antibiotics within 4hrs is decreased by 4%
**Gaieski et al (2017)**	Retrospective cohort	USA, trauma centre Jan 2005-Feb 2010	2913 adults, mean age: 57yrs, 47% female	Patients with sepsis	IVF & antibiotic delivery within 1hr	high occupancy rate delayed antibiotic and IVF delivery >3hrs, OR 0.77	Delay in daytime shift is >1hr greater than night time; OR 0.73 (95%CI 0.59 to 0.64) (95% CI 0.51 to 0.81) respectively

						(95% CI 0.61 to 0.96)	
**Peltan et al (2019)**	Retrospective cohort	USA, 2 trauma centres & 2 community hospitals Jul 2013-Sep 2015	3572 adults, mean age: 64yrs, 54% female	Patients with sepsis	Door-to-antibiotics within 1hr	46% received in >3hrs; 61% within 3hrs. OR 0.88 (95% CI 0.86 to 0.91)	each 10% increase in occupancy rate is linked with a 4 minute increase
**Pines et al (2007)**	Retrospective cohort	USA, academic centre Jan 2003-April 2005	694 adults, mean age: 57yrs, 50% female	Patients with CAP	Door-to-antibiotics within 4hrs	only 44% received within 4hrs, OR 1.05 (95% CI 1.01-1.10)	7 mins delay in antibiotic delivery for each additional patient arrival
**Pines & Hollander (2008)**	Retrospective cohort	USA, academic centre April 2005-Sep 2006	13758 adults, mean age: 39yrs, 64% female	Patients with severe pain	Analgesics initiation within 1hr	occupancy rate associated with 59% experienced delay from	74 mins delay from triage to treatment & 20 mins delay from placement room to treatment

						triage to analgesia while 20% delay from placement room to analgesia; OR 1.18 (95% CI 1.15-1.21) OR 1.06 (95% CI 1.04-1.08) respectively	
**Pines et al (2010)**	Retrospective cohort	USA, academic centre & community hospital July 2003-Feb 2007	5616 adults, mean age: 44yrs, 57% female	Patients with severe back pain	Time-to-analgesia within 1hr	81% experienced delay >1hr from triage to treatment, 67% from placement room to	delays in the academic site is higher, P <0.001

						treatment (130mins vs 86mins)	
**Sikka et al (2010)**	Retrospective cohort	USA, community hospital Oct 2007-Feb 2008	334 adults	Patients with CAP	Antibiotic delivery within 4hrs	high occupancy rate delayed antibiotic delivery >4hrs, OR 0.31 (95% CI 0.13-0.75) P = 0.008	increased occupancy rate is associated with antibiotic delay > 4hrs
**Mills et al (2009)**	Prospective cohort	USA, academic centre April 2004-Jan 2005	976 adults, mean age: 41yrs, 65% female	Patients with acute abdominal pain	Time-to-analgesia within 1hr	70% experienced delay > 1hr from triage while 49% from room placement	time-to-analgesia from triage is longer than that of room placement to analgesia, P= 0.0001

**Pines et al (2006)**	Cross- sectional	USA, 24 academic centres Jan-Dec 2004	50537 adults	Patients with pneumonia	Time-to-antibiotics within 4hrs	increased LOS delayed antibiotic delivery > 4hrs (-0.44, P= 0.04)	only 59% received within 4hrs
**Linden et al (2016)**	Retrospective cohort	Netherlands, medical centre Jan 2009-Dec 2013	39110 adults, mean age: 61yrs, 49% male	Patients with general pain	Door-to-needle	higher delay during crowding time to noncrowding (11.7% vs 7.3%), P= <0.001	higher LOS is associated with > 4hrs delay during crowding time compared to noncrowding (42.5% vs 34%)

CAP: community-acquired pneumonia, IVF: intravenous fluids, OR: odd ratio, CI: confidence interval, LOS: length of stay [illegible]

Table S2. CASP checklist for cohort studies

Appraisal Questions/Authors	Fee et al (2007)	Gaieski et al (2017)	Peltan et al (2019)	Pines et al (2007)	Pines & Hollander (2008)	Pines et al (2010)	Sikka et al (2010)	Mills et al (2009)	Pines et al (2006)	Linden et al(2016)
Did the study address a clearly focused issue?	Yes	Yes	Yes	Yes	Yes	Yes	Yes	Yes	Yes	Yes
Was the cohort recruited in an acceptable way?	Yes	Yes	Yes	Yes	Yes	Yes	Yes	Yes	Yes	Yes
Was the exposure accurately measured to	Yes	Yes	Yes	Yes	Yes	Yes	Yes	Yes	Yes	Yes

minimise bias?										
Was the outcome accurately measured to minimise bias?	Yes	Yes	Yes	Yes	Yes	Yes	Yes	Yes	Yes	Yes
Have the authors identified all confounding factors?	Can't tell	No	Can't tell	Can't tell	No	Can't tell	No	Can't tell	No	Can't tell
Have they taken account of the confounding factors in the	Yes	No	Can't tell	Yes	Can't tell	Yes	Can't tell	Yes	No	Can't tell

design and/or analysis?										
Was the follow up of subjects complete enough?	Can't tell	Yes	Yes	Yes	Yes	No	Yes	Can't tell	Yes	Yes
Was the follow-up of subjects long enough?	No	Can't tell	Can't tell	Can't tell	Can't tell	Can't tell	Can't tell	Can't tell	No	Yes
What are the results of the study?	Increase in patient volume reduced timely antibiotic	ED crowding increase time for critical sepsis	ED overcrowding increase sepsis antibiotic delay	ED crowding delayed the antibiotic receipt	ED overcrowding delay treatment and led to non-	The overcrowding delayed administration of pain medication	High occupancy rate in the ED increase treatment failure by	Time to analgesia is impacted by ED overcrowding	Administration of antibiotics is affected by ED overcrowding	High occupancy rate delayed treatment time

	therapy in the ED	therapies and initiation of care protocols		for ED patients	treatment of patients	on analgesia	over 4hours			
How precise are the results?	Highly precise (95% CI used)	Highly precise (95% CI used)	Highly precise (95% CI used)	Highly precise (95% CI used)	Highly precise (95% CI used)	Can't tell	Highly precise (95% CI used)	Highly precise (95% CI used)	Can't tell	Can't tell
Do you believe the results?	Yes	Yes	Yes	Yes	Yes	Yes	Yes	Yes	Yes	Yes
Can the results be applied to the local problem?	Yes	Yes	Yes	Yes	Yes	Yes	Yes	Yes	Yes	Yes
Do the results of this study fit	Yes	Yes	Yes	Yes	Yes	Yes	Yes	Yes	Yes	Yes

with other available evidence?										
What are the implications of this study for practice?	Administrators should build quality targets to manage the impact of ED volume	ED should implement frameworks for optimizing the delivery of time-sensitive therapies to patients needing	Hospitals should develop ways to enhance sepsis antibiotic administration in the ED crowding periods	Examining patient and provider-level effects should indicate ED crowding to manage time-	ED crowding should be improved to enhance healthcare system functioning and reduce crowding and enhance pain	Taking static measures for ED crowding should indicate the occupancy rate and influence timelines and quality of care	Validity of occupancy rate in the ED should increase ED administration	Care providers should emphasise on pain management & control prior to identifying the disease	Organisational measures to reduce inpatient boarding, such as rapid lab & Xray investigations may reduce ED overcrowding	Hospital measures to mitigate elapsed time from triage to therapy initiation must take place

	emergen cy services	sensitiv e care	manageme nt	

Table S3. JBI checklist for cross-sectional studies

Checklist questions	Fee et al (2007)	Pines et al (2006)
Were the criteria for inclusion in the sample clearly defined?	√	√
Were the study subjects and the setting described in detail?	√	√
Was the exposure measured in a valid and reliable way?	√	√
Were objective, standard criteria used for measurement of the condition?	√	√
Were confounding factors identified?	Unclear	No
Were strategies to deal with confounding factors stated?	Unclear	No
Were the outcomes measured in a valid and reliable way?	√	√
Was appropriate statistical analysis used?	√	√

Table S4. Delay of analgesia as recorded by median time

Study	Triage	Room placement	Percentage difference
Mills et al (2009)	107 mins	61 mins	55%
Pines et al (2008)	74 mins	23 mins	105%
Pines et al (2010)	130 mins	86 mins	41%
